# Tobacco smoking is associated with DNA methylation of diabetes susceptibility genes

**DOI:** 10.1007/s00125-016-3872-0

**Published:** 2016-01-29

**Authors:** Symen Ligthart, Rebecca V. Steenaard, Marjolein J. Peters, Joyce B. J. van Meurs, Eric J. G. Sijbrands, André G. Uitterlinden, Marc J. Bonder, BIOS consortium, Albert Hofman, Oscar H. Franco, Abbas Dehghan

**Affiliations:** Department of Epidemiology, Erasmus University Medical Center, P. O. Box 2040, 3000 CA Rotterdam, the Netherlands; Department of Internal Medicine, Erasmus University Medical Center, Rotterdam, the Netherlands; The Netherlands Genomics Initiative-sponsored Netherlands Consortium for Healthy Aging (NGI-NCHA), Leiden/ Rotterdam, the Netherlands; Department of Genetics, University of Groningen, University Medical Centre Groningen, Groningen, the Netherlands

**Keywords:** DNA methylation, Gene expression, Tobacco smoking, Type 2 diabetes

## Abstract

**Aims/hypothesis:**

Tobacco smoking, a risk factor for diabetes, is an established modifier of DNA methylation. We hypothesised that tobacco smoking modifies DNA methylation of genes previously identified for diabetes.

**Methods:**

We annotated CpG sites available on the Illumina Human Methylation 450K array to diabetes genes previously identified by genome-wide association studies (GWAS), and investigated them for an association with smoking by comparing current to never smokers. The discovery study consisted of 630 individuals (Bonferroni-corrected *p* = 1.4 × 10^−5^), and we sought replication in an independent sample of 674 individuals. The replicated sites were tested for association with nearby genetic variants and gene expression and fasting glucose and insulin levels.

**Results:**

We annotated 3,620 CpG sites to the genes identified in the GWAS on type 2 diabetes. Comparing current smokers to never smokers, we found 12 differentially methylated CpG sites, of which five replicated: cg23161492 within *ANPEP* (*p* = 1.3 × 10^−12^); cg26963277 (*p* = 1.2 × 10^−9^), cg01744331 (*p* = 8.0 × 10^−6^) and cg16556677 (*p* = 1.2 × 10^−5^) within *KCNQ1* and cg03450842 (*p* = 3.1 × 10^−8^) within *ZMIZ1*. The effect of smoking on DNA methylation at the replicated CpG sites attenuated after smoking cessation. Increased DNA methylation at cg23161492 was associated with decreased gene expression levels of *ANPEP* (*p* = 8.9 × 10^−5^). rs231356-T, which was associated with hypomethylation of cg26963277 (*KCNQ1*), was associated with a higher odds of diabetes (OR 1.06, *p* = 1.3 × 10^−5^). Additionally, hypomethylation of cg26963277 was associated with lower fasting insulin levels (*p* = 0.04).

**Conclusions/interpretation:**

Tobacco smoking is associated with differential DNA methylation of the diabetes risk genes *ANPEP*, *KCNQ1* and *ZMIZ1*. Our study highlights potential biological mechanisms connecting tobacco smoking to excess risk of type 2 diabetes.

**Electronic supplementary material:**

The online version of this article (doi:10.1007/s00125-016-3872-0) contains peer-reviewed but unedited supplementary material, which is available to authorised users.

## Introduction

In the last decade, genome-wide association studies (GWAS) have been conducted in order to identify DNA sequence variants for a wide range of diseases including type 2 diabetes [1–3]. These GWAS have successfully identified numerous single-nucleotide polymorphisms (SNPs) located in and near genes that may be key in the development of type 2 diabetes. Up to now, a total number of 88 genetic loci have been identified for type 2 diabetes [4].

Tobacco smoking is associated with an increased risk of type 2 diabetes [5]. Several biological mechanisms have been proposed through which smoking may have an effect on the development of diabetes, including inflammation and the effect of nicotine on insulin resistance [6]. However, the exact molecular mechanisms connecting smoking to an increased risk of diabetes remain largely unknown. Previous research has established that tobacco smoking has an important role in DNA methylation, the epigenetic mechanism of attachment of a methyl group to a nucleotide [7–9]. DNA methylation has several functions in the human genome including the regulation of gene expression and maintenance of genome stability [10]. In line with this, previous studies have suggested DNA methylation as a potential pathway in the association between tobacco smoking and an increased risk of diabetes [11].

We hypothesised that tobacco smoking changes DNA methylation of susceptibility loci identified in GWAS for type 2 diabetes. We therefore investigated the association between DNA methylation in whole blood at loci identified for type 2 diabetes through GWAS and current tobacco smoking in a Dutch population-based cohort study. Furthermore, we investigated the potential effect of DNA methylation on the expression of genes near to the identified methylation sites.

## Methods

### Study population

The study was conducted using data from the Rotterdam Study; the design of the Rotterdam Study has been described elsewhere [12]. In brief, in 1990 all inhabitants living in the neighbourhood of Ommoord in Rotterdam, the Netherlands, aged 55 years and over, were invited to participate (RS-1). In 2000, the cohort was extended with 3,011 participants who had reached the age of 55 years or who were aged 55 years and over and had moved into the research area (RS-2). In 2006, a third cohort of 3,934 participants aged 45 years and older was initiated (RS-3). The discovery panel consisted of 630 non-diabetic participants in the first visit of RS-3 (diabetes was defined as a serum glucose level ≥ 7.0 mmol/l or the use of glucose-lowering medication) of a random subset of 747 individuals of European descent with DNA methylation data available. We sought replication of the identified CpG sites in a set of 674 non-diabetic participants from the third visit of RS-2 and the second visit of RS-3. The individuals in the replication study did not participate in the discovery study. The Rotterdam Study has been approved by the medical ethics committee according to the Population Screening Act: Rotterdam Study, executed by the Ministry of Health, Welfare and Sports of the Netherlands. All participants in the present analysis provided written informed consent to participate and to obtain information from their treating physicians.

### Data collection

Data on tobacco smoking was collected during home interviews. Participants were asked about past and present cigarette, cigar and pipe smoking behaviour and were then categorised into current, former and never tobacco smokers. We asked current smokers about the age at which they started smoking and the number of cigarettes that they smoked per day. Former smokers were asked at what age they ceased smoking. Five of the participants had missing smoking status and were therefore excluded from any analysis. During the visit to the centre, weight and height were measured with the participant in standing position and wearing normal clothes. BMI was calculated as height in metres by weight in kilograms squared. All participants had blood samples taken during the visit to quantify DNA methylation, messenger RNA (mRNA) expression levels, DNA sequence variants and other blood measurements.

### DNA methylation data

DNA was extracted from whole peripheral blood (stored in EDTA tubes) by standardised salting out methods. Genome-wide DNA methylation levels were measured using the Illumina Human Methylation 450K array [13] (Illumina, San Diego, CA, USA). In short, samples (500 ng of DNA per sample) were first treated with bisulfite using the Zymo EZ-96 DNA-methylation kit (Zymo Research, Irvine, CA, USA). Next, samples were hybridised to the arrays according to the manufacturers’ protocols. The methylation percentage of a CpG site was reported as a β value ranging between 0 (no methylation) and 1 (full methylation). Processing of the Rotterdam Study DNA methylation samples was performed at the Genetic Laboratory of Internal Medicine, Erasmus University Medical Centre, Rotterdam.

Quality control of the samples was carried out using Genome Studio (v2011.1, methylation module version 1.9.0; Illumina). In the discovery panel, a total number of 16 samples were removed: seven had a sample call rate below 99%; five had incomplete bisulfite conversion and four had sex changes. In the replication set, all samples passed the quality control based on the first two principal components obtained using principal component analysis (PCA), and no sex swaps were detected. Further quality control of the probes was done based on the detection *p* value calculated with Genome Studio. Probes with a detection *p* value of more than 0.01 in more than 1% of the samples were excluded. Additionally, sample-level quality control was performed using MethylAid (https://bioconductor.org/packages/release/bioc/html/MethylAid.html) [14]. This resulted in a total set of 474,528 probes that were normalised using the Dasen option of the WateRmelon R-package (https://www.bioconductor.org/packages/release/bioc/html/wateRmelon.html) [15].

### mRNA expression data

Whole blood was collected (PAXGene Tubes; Becton Dickinson, Erembodegem, Belgium) and total RNA was isolated (PAXGene Blood RNA kits; Qiagen, Venlo, the Netherlands). To ensure the constant high quality of the RNA preparations, all RNA samples were analysed using the Labchip GX (Caliper, Hopkinton, MA, USA) according to the manufacturer’s instructions. Samples with an RNA quality score of more than 7 were amplified and labelled (TotalPrep RNA; Ambion, Austin, TX, USA) and hybridised to the Illumina HumanHT12v4 Expression Beadchips (Illumina) as described by the manufacturer’s protocol. Processing of the Rotterdam Study RNA samples was performed at the Genetic Laboratory of Internal Medicine, Erasmus University Medical Centre, Rotterdam. The RS-3 expression dataset is available at GEO (Gene Expression Omnibus) public repository under the accession GSE33828: 881 samples are available for analysis.

Illumina gene expression data was quantile-normalised to the median distribution and subsequently log_2_-transformed. The probe and sample means were centred to zero. Genes were declared significantly expressed when the detection *p* values calculated by GenomeStudio were less than 0.05 in more than 10% of all discovery samples, which added to a total number of 21,238 probes. Quality control was carried out using the eQTL-mapping pipeline (https://github.com/molgenis/systemsgenetics/tree/master/eqtl-mapping-pipeline) [16]. We only analysed probes that uniquely mapped to the human genome build 37 and represented gene mRNA expression [17].

### Selection of methylation sites

A recent review summarising findings from all diabetes GWAS was used to compile a list of variants significantly associated with diabetes (88 variants) [4]. Next, the list of 88 variants was extended with polymorphisms in linkage disequilibrium (*R*^2^ > 0.8) in the HapMap panel and within 500 kb using the SNAP Proxy Search tool (https://broadinstitute.org/mpg/snap/ldsearch.php; accessed 1 October 2015). The final list included 890 SNPs, which were tested for in-gene variants and effects on expression of a gene within 1 Mb as found in a large publically available blood *cis*-expression-quantitative trait loci (*cis*-eQTL) database (false discovery rate [FDR] <0.05) [16]. We identified 525 SNPs that were in-gene (mapping to 72 unique genes) and 316 SNPs with an eQTL effect (mapping to 50 unique genes). The final number of unique genes was 111. The methylation probes within and near these diabetes-related genes as provided by Illumina were included in the analysis. We excluded probes from the Infinium HD methylation SNP list with a minor allele frequency above 1% as provided by Illumina, since variations in these SNPs can cause bias in the methylation measurement [18]. We further excluded known cross-reactive probes, since they can introduce bias in the results [19]. In total, we included 3,620 CpG sites in the analyses.

### Statistical analysis

The characteristics of the discovery and replication populations were compared between current and never smokers using IBM SPSS Statistics version 21.0.0.1 (IBM, Armonk, NY, USA). The *p* values were calculated using independent sample *t* tests for continuous variables and *χ*^2^-square tests for dichotomous variables.

The 3,620 methylation probes were tested for association with tobacco smoking using a linear mixed model with the LME4 package in R version 3.1.0 with Dasen-normalised β values of the CpG sites as outcome measure (https://cran.r-project.org/web/packages/lme4/index.html) [20]. Extreme outliers (>4 SD from the mean and >4SD from the before last) in the DNA methylation values were excluded. We first compared current smokers with never smokers and then performed a sensitivity analysis on the identified CpG sites comparing former smokers with never smokers. Covariates were selected based on known association with DNA methylation. The selected covariates with fixed effects were age, sex and BMI [21–24]. Houseman-estimated white blood cell proportions were used as fixed effects to correct for cell mixture distribution [25]. Array number and position on array were added in the model as covariates with random effects to correct for batch effects. We corrected for multiple testing using a robust Bonferroni-corrected *p* value of 1.4 × 10^−5^ as the threshold for significance (0.05 / 3,620 probes).

The probes identified in the discovery analysis were tested for replication in the independent samples from the Rotterdam Study. We used identical models with the addition of cohort (RS-2 or RS-3) as a variable in the model to adjust for a potential cohort effect. A Bonferroni-corrected *p* value of 0.05 divided by the number of significant findings in the discovery study was used as a threshold of significant replication.

The replicated probes were further tested with total pack-years in the current smokers to test the association between tobacco smoking and cumulative exposure to smoking. We further investigated the association between the replicated probes and time since cessation in former smokers to study the change in methylation after smoking cessation. To decrease the possibility of confounding in our association, we further adjusted the model in a second analysis for other possible confounders and mediators. This analysis included total cholesterol, HDL-cholesterol, triacylglycerol levels (natural log-transformed), systolic blood pressure, daily alcohol intake and C-reactive protein levels (natural log-transformed).

### Functional analysis

Since DNA methylation may have an effect on gene expression, we tested the association between DNA methylation and mRNA expression levels of nearby genes (*cis*) within 500 kb of the replicated CpG sites (250 kb upstream and downstream of the CpG location). First, residuals for mRNA expression were created after regressing out the measured cell counts (granulocytes, lymphocytes, monocytes, platelets and erythrocytes), fasting state, RNA quality score, plate number, age and sex on the mRNA expression levels using a linear mixed model. We then created residuals for DNA methylation regressing out the measured white blood cells, age, sex, array number and position on array on the Dasen-normalised β values of the CpG sites using a linear mixed model. The residuals of the mRNA expression levels and the residuals of the Dasen-normalised β values of the CpG sites were tested for association using a linear regression model.

We also studied the association between the replicated CpG sites and serum measures of fasting glucose and insulin combining both the discovery and replication samples. Serum glucose and insulin were measured using standard laboratory techniques. The models were adjusted for the same covariates as in the main analyses, with the addition of smoking category. Serum insulin was natural log-transformed. A Bonferroni-corrected *p* value for five tests was used. Furthermore, we searched for genetic variants (methylation quantitative trait loci [met-QTLs]) associated with the replicated methylation sites in the publicly available data from the paper by Grundberg et al [26]. Significant met-QTLs were then tested for an association with type 2 diabetes in the publicly available data from the DIAGRAM consortium, using a Bonferroni-corrected *p* value of 0.01 (0.05 / 5 met-QTLs) [3].

## Results

A total of 630 participants were included in the discovery study. Clinical characteristics of the study population by smoking category are listed in Table 1. The participants were on average 59.5 ± 8.0 years old and 45% were men. The samples consisted of 175 current smokers, 184 never smokers and 271 former smokers. On average, current smokers had lower HDL-cholesterol, higher triacylglycerol and serum C-reactive protein than never smokers. Also alcohol consumption was higher in current smokers than in former smokers or never smokers. In the replication population, 68 individuals were current smokers, 238 were never smokers and 368 were former smokers. Clinical characteristics of the replication population can be found in electronic supplementary material (ESM) Table 1.

**Table 1:** Baseline characteristics of the study population according to smoking status

Characteristic	Total	Current	Former	Never	*p* value^a^
*N*	630	175	271	184	
Age, years	59.5 ± 8.0	57.9 ± 6.6	60.9 ± 8.5	59.0 ± 8.1	0.16
Male sex (%)	283 (45)	85 (49)	126 (47)	72 (39)	0.07
BMI, kg/m^2^	27.4 ± 4.5	26.7 ± 4.4	27.6 ± 4.3	27.6 ± 4.8	0.07
Fasting glucose, mmol/l	5.35 ± 0.55	5.33 ± 0.58	5.40 ± 0.55	5.30 ± 0.52	0.65
Systolic blood pressure, mmHg	138.5 ± 63.0	136.4 ± 60.4	139.7 ± 67.4	138.7 ± 58.8	0.71
Diastolic blood pressure, mmHg	88.0 ± 65.0	86.0 ± 62.1	89.0 ± 9.8	88.4 ± 60.3	0.71
Total cholesterol, mmol/l	5.60 ± 1.03	5.60 ± 1.07	5.62 ± 1.01	5.56 ± 1.02	0.72
HDL-cholesterol, mmol/l	1.41 ± 0.40	1.34 ± 0.39	1.44 ± 0.41	1.44 ± 0.37	0.01
Triacylglycerol, mmol/l	1.45 ± 0.81	1.62 ± 1.02	1.39 ± 0.62	1.40 ± 0.81	0.02
C-reactive protein, mg/l	2.55 ± 4.74	3.17 ± 7.03	2.52 ± 3.54	2.03 ± 3.31	0.05
Alcohol consumption, g/day	18.3 ± 11.0	19.4 ± 12.7	19.0 ± 10.9	16.1 ± 9.3	0.006
Fasting^b^, yes (%)	628 (100)	173 (99)	271 (100)	184 (100)	0.15

Data are mean ± SD or *n* (%)

^a^Current vs never smokers

^b^The participants who provided blood after an overnight fast

After correction for multiple testing (*p* = 1.4 × 10^−5^), we identified 12 differentially methylated CpG sites when comparing current smokers to never smokers in the discovery study (Table 2; results for all probes are presented in ESM Table 2). The 12 differentially methylated CpG sites were located within eight genes. The most significant finding was cg23161492 located within the gene *ANPEP* on chromosome 15 (*p* = 1.3 × 10^−12^). On chromosome 11, four CpG sites located within the gene *KCNQ1* were significantly associated with current tobacco smoking (cg26963277, *p* = 1.2 × 10^−9^; cg13428066, *p* = 5.8 × 10^−6^; cg01744331, *p* = 8.0 × 10^−6^; cg16556677, *p* = 1.2 × 10^−5^). Within the gene *ZMIZ1* on chromosome 10, two CpG sites were significant differentially methylated between current and never smokers (cg03450842, *p* = 3.1 × 10^−8^; cg21344746, *p* = 6.6 × 10^−6^). In addition, we identified CpG sites in and near *INPP5E*, *NDUFS5*, *FCHSD2*, *PBX4* and *TCF19* that were differentially methylated in current smokers compared with never smokers.

**Table 2:** Significant associations between current vs never tobacco smoking and methylation of diabetes genes

CpG site	Chromosome	Position Hg19	Discovery			Replication			Gene
			β	SE	*p* value	β	SE	*p* value	
cg23161492	15	90357202	−0.044	0.006	1.3 × 10^−12^	−0.045	0.006	3.4 × 10^−11^	*ANPEP*
cg26963277	11	2722407	−0.026	0.004	1.2 × 10^−9^	−0.034	0.004	3.3 × 10^−14^	*KCNQ1*
cg03450842	10	80834947	−0.017	0.003	3.1 × 10^−8^	−0.030	0.004	2.2 × 10^−12^	*ZMIZ1*
cg14024579	9	139332845	−0.022	0.004	1.1 × 10^−7^	−0.015	0.006	0.01	*INPP5E*
cg14656441	1	39500070	0.026	0.005	1.5 × 10^−6^	0.016	0.008	0.05	*NDUFS5*
cg13912027	11	72759293	0.022	0.005	2.1 × 10^−6^	−0.001	0.006	0.89	*FCHSD2*
cg00591868	19	19729048	−0.015	0.003	4.6 × 10^−6^	−0.003	0.005	0.51	*PBX4*
cg13428066	11	2677768	0.015	0.003	5.8 × 10^−6^	0.007	0.006	0.28	*KCNQ1*
cg21344746	10	80831230	0.016	0.004	6.6 × 10^−6^	0.001	0.005	0.82	*ZMIZ1*
cg16095155	6	31127863	−0.013	0.003	7.2 × 10^−6^	−0.007	0.004	0.12	*TCF19*
cg01744331	11	2722358	−0.013	0.003	8.0 × 10^−6^	−0.025	0.003	7.4 × 10^−12^	*KCNQ1*
cg16556677	11	2722401	−0.015	0.003	1.2 × 10^−5^	−0.027	0.004	3.9 × 10^−10^	*KCNQ1*

Adjusted for age, sex, BMI, Houseman-estimated white blood cell proportions and batch effects

Bonferroni-corrected threshold for significance: 0.05/3,620 = 1.4 × 10^−5^

**Fig. 1:**
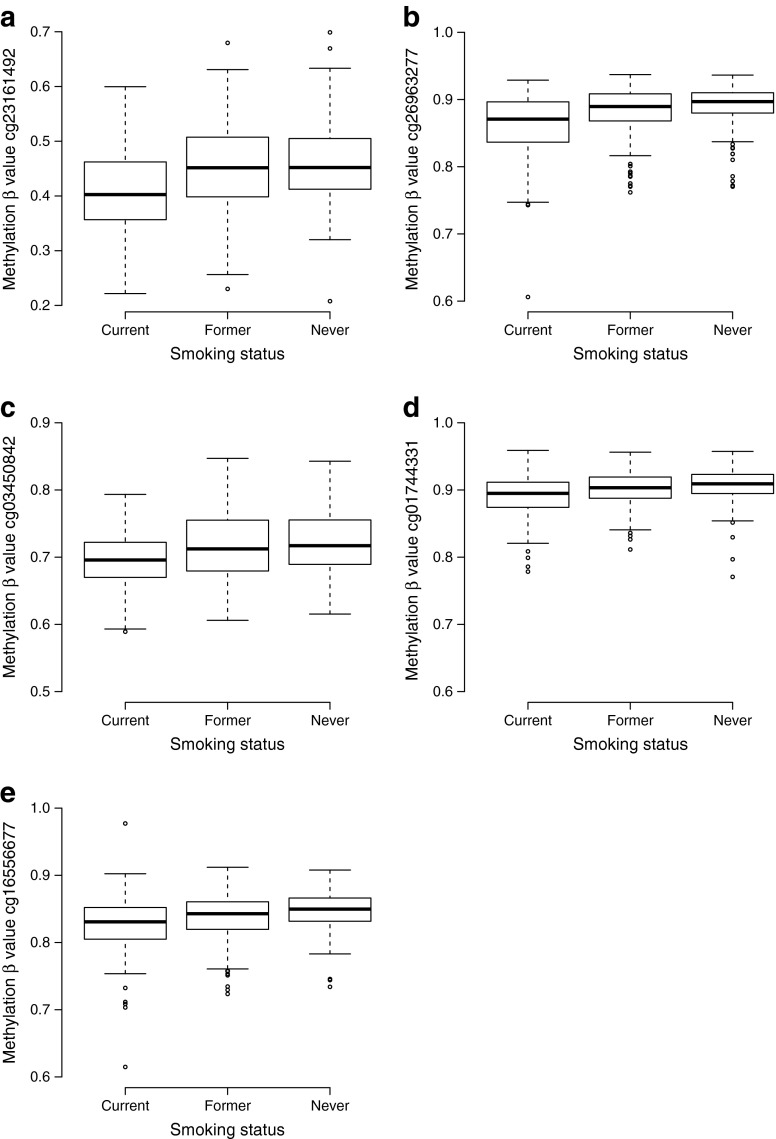
Boxplots depicting the methylation values in the replicated CpG sites (cg23161492 [**a**], cg26063277 [**b**], cg03450842 [**c**], cg01744331 [**d**], cg16556677 [**e**]) in current, former and never smokers. The bold horizontal lines represent the median methylation values, the box represents the interquartile range, the whiskers extend to 1.5 times the interquartile range or the most extreme value, and the circles represent a participant’s unique methylation value

We attempted replication of the 12 differentially methylated CpG sites from the discovery study in 674 independent participants of the second and third cohort of the Rotterdam Study. We used a *p* value of 4.2 × 10^−3^ (0.05 / 12) as a threshold of significant replication. We significantly replicated the five CpG sites cg23161492 (*ANPEP*), cg26963277 (*KCNQ1*), cg03450842 (*ZMIZ1*), cg01744331 (*KCNQ1*) and cg16556677 (*KCNQ1*) (Table 2). Furthermore, the replicated associations were robust to further adjustment for possible confounders including systolic blood pressure, total cholesterol, HDL-cholesterol, triacylglycerol, alcohol consumption and C-reactive protein (ESM Table 3). Boxplots of replicated probe β values per smoking category are presented in Fig. 1. When we adjusted the effect of the top signal within the *KCNQ1* gene (cg26963277) for the second (cg01744331) or third (cg16556677) signal within *KCNQ1*, cg26963277 was associated with current smoking, whereas cg01744331 and cg16556677 did not show an association (*p* = 0.84 and 0.35, respectively).

To study the effect of smoking cessation on the replicated CpG sites, we compared former smokers with never smokers and tested the association between time since smoking cessation and DNA methylation. DNA methylation at the five CpG sites were not differentially methylated when former smokers were compared with never smokers (Table 3). Methylation at cg23161492 (*p* = 2.6 × 10^−6^), cg26963277 (*p* = 2.1 × 10^−4^), cg01744331 (*p* = 5.1 × 10^−5^) and cg16556677 (*p* = 1.2 × 10^−3^) was associated with time since smoking cessation. Additionally, methylation at the CpG sites cg23161492, cg26963277, cg03450842 and cg01744331 was associated with cumulative exposure to tobacco smoking.

**Table 3:** Association between CpG sites and former smokers compared with never smokers, time since smoking cessation and cumulative smoking exposure in pack-years

CpG site	Gene	Former vs never smokers		Cessation time		Pack-years	
		β (SE)	*p* value	β^a^ (SE)	*p* value	β^a^ (SE)	*p* value
cg23161492	*ANPEP*	−0.007 (0.006)	0.24	0.014 (0.003)	2.6 × 10^−6^	−0.007 (0.002)	2.8 × 10^−3^
cg26963277	*KCNQ1*	−0.006 (0.003)	0.05	0.006 (0.002)	2.1 × 10^−4^	−0.006 (0.002)	9.0 × 10^−4^
cg03450842	*ZMIZ1*	−0.005 (0.002)	0.06	0.002 (0.001)	0.20	−0.003 (0.001)	1.6 × 10^−3^
cg01744331	*KCNQ1*	−0.003 (0.002)	0.21	0.005 (0.001)	5.1 × 10^−5^	−0.004 (0.001)	1.1 × 10^−4^
cg16556677	*KCNQ1*	−0.007 (0.003)	7.1 × 10^−3^	0.005 (0.001)	1.2 × 10^−3^	−0.003 (0.001)	0.05

Adjusted for age, sex, BMI, white blood cell counts and batch effects. Bonferroni corrected

^a^β represents change in methylation per 10 years since smoking cessation and per 10 pack-years.

In the 630 individuals from the discovery panel, six genes out of 20 candidates were significantly expressed in the analysed whole-blood samples. The 12 methylation expression combinations are shown in ESM Table 4. The *p* value threshold for association was 4.2 × 10^−3^ (0.05/12 tests). Increased methylation at cg23161492 was negatively associated with gene expression levels of *ANPEP* (*p* = 8.9 × 10^−5^) (ESM Fig. 1).

We observed a putative effect of the CpG site cg26963277 with fasting serum insulin (effect: 0.004, *p* = 0.04). Results for the associations between all replicated CpG sites and serum fasting glucose and insulin are presented in ESM Table 5.

We identified a significant met-QTL for all replicated CpG sites, except cg0345084 (ESM Table 6). The T allele of the SNP rs231356 was associated with lower methylation of both cg26963277 and cg01744331 (*KCNQ1*). Also, the T allele of the SNP rs231356 was associated with an increased odds of type 2 diabetes (OR 1.06, *p* = 1.3 × 10^−5^).

## Discussion

Our findings suggest that tobacco smoking is associated with differential methylation of CpG sites within the type 2 diabetes risk genes *ANPEP*, *KCNQ1* and *ZMIZ1*. The associations were robust to adjustment for potential confounders and the effect of tobacco smoking appeared to be reversible after smoking cessation. In addition, methylation within *ANPEP* was significantly associated with gene expression levels of *ANPEP*. Methylation at *KCNQ1* was associated with fasting insulin levels and genetic data supported a role for methylation at *KCNQ1* in the development of diabetes. This study provides further insight into potential biological mechanisms underlying the association between tobacco smoking and an excess risk of type 2 diabetes.

In contrast to the findings for current vs never smokers, we found similar DNA methylation levels at the replicated CpG sites when comparing former smokers to never smokers. Furthermore, four significant CpG sites were associated with time since smoking cessation, suggesting a return after smoking cessation to DNA methylation levels similar to never smokers. This is in agreement with previous studies investigating the role of smoking cessation in DNA methylation [7, 27, 28]. DNA methylation may return to levels similar to never smokers at some sites, while other sites stay differentially methylated. Our results are in agreement with a potential beneficial effect of smoking cessation on DNA methylation at risk loci for diabetes. Furthermore, at four CpG sites we observed a dose-dependent effect of smoking underscoring the importance of cumulative tobacco exposure over time.

We identified three CpG sites within intron 11 of *KCNQ1* (potassium channel, voltage gated KQT-like subfamily Q, member 1) that were differentially methylated in current smokers compared with never smokers. Previous studies have reported differential DNA methylation at the *KCNQ1* locus in pancreatic islets and adipose tissue of diabetes cases and non-diabetes controls [29, 30]. Adjustment analyses suggested that cg26963277 is the driving CpG site associated with current smoking at this locus. Furthermore, we found the met-QTL (rs231356) for cg26963277 to be associated with the risk of diabetes. More specifically, the T allele of rs231356 is associated with lower methylation of cg26963277 and an increased odds of type 2 diabetes. In agreement with this observation, tobacco smoking lowers methylation at cg26963277 and is associated with an increased risk of diabetes. Additionally, our data suggest an association between cg26963277 and fasting insulin levels: increased methylation was putatively associated with increased fasting insulin levels. Although we did not observe an association between DNA methylation at cg26963277 and expression of *KCNQ1*, our results provide evidence that smoking may increase the risk of diabetes through decreased methylation at *KCNQ1* and a subsequent decrease in fasting insulin levels.

Further, current tobacco smoking was associated with a 4.4% decrease in methylation at cg23161492 located near the 5′ untranslated region (UTR) of *ANPEP* and this decreased methylation was correlated with increased gene expression levels of *ANPEP. ANPEP* encodes the protein alanine aminopeptidase, a widely expressed enzyme involved in various cellular processes including cell proliferation, differentiation and apoptosis [31]. The observation that current smoking, which increases the risk of type 2 diabetes, may lead to higher gene expression levels of *ANPEP* is in line with the observation of Locke and colleagues [32]. The risk allele of the SNP rs2007084, identified by the DIAGRAM consortium, is also associated with increased gene expression of *ANPEP* in islet cells [32]. This suggests that increased expression of *ANPEP* leads to an increased risk of type 2 diabetes. The observation that DNA sequence variation and DNA methylation at this locus is associated with increased expression levels of *ANPEP* suggests a role for *ANPEP* in the pathogenesis of type 2 diabetes, rather than the gene *AP3S2* proposed by prior GWAS [3].

We further identified the CpG cg03450842, near the 5′ UTR of *ZMIZ1*, to be differentially methylated in smokers compared with never smokers. The CpG cg03450842 has been identified previously to be associated with smoking [11]. Unfortunately, we had no expression data available in our samples for this gene and could therefore not study the effect of methylation at cg03450842 on gene expression of *ZMIZ1*.

The strength of the current study is the large sample size with available data on DNA methylation, gene expression and genetic variants, which allowed detailed investigation of the interrelationship between tobacco smoking, DNA methylation and gene expression. A limitation of the current work is the use of whole-blood samples for the quantification of DNA methylation and gene expression. As both methylation and expression may be tissue specific, we might have overlooked potential associations between tobacco smoking and differential methylation of diabetes-related genes in other tissues (e.g. liver, fat, pancreas or muscle tissue). Furthermore, observed associations may not be generalisable to other tissues. Another limitation is the challenge of gene annotation in GWAS. GWAS locate DNA sequence variants for phenotypes, but the underlying causal gene might be difficult to designate. To minimise this problem we limited our analysis to genes annotated to in-gene variants and known *cis*-eQTL effects. Therefore the diabetes risk genes selected in our study are more plausible as being the causal gene for diabetes.

In summary, our study suggests an effect of tobacco smoking on DNA methylation of the diabetes-related genes *ANPEP*, *KCNQ1* and *ZMIZ1*. Our study provides further insight into potential mechanisms linking tobacco smoking to an excess risk of type 2 diabetes.

## Electronic supplementary material

Below is the link to the electronic supplementary material.

ESM Fig. 1 (PDF 94 kb)

ESM Table 1 (PDF 21 kb)

ESM Table 2 (XLSX 160 kb)

ESM Table 3 (PDF 7 kb)

ESM Table 4 (PDF 21 kb)

ESM Table 5 (PDF 7 kb)

ESM Table 6 (PDF 20 kb)

## Abbreviations

GWAS: Genome-wide association studies
met-QTLs: Methylation quantitative trait loci
SNP: Single-nucleotide polymorphism
UTR: Untranslated region
