# Supplementary material for: Tobacco smoking is associated with DNA methylation of diabetes susceptibility genes
Source: Diabetologia. 2016 Jan 29;59:998–1006. doi: 10.1007/s00125-016-3872-0 (PMC4826423; doi:10.1007/s00125-016-3872-0)
Supplement: Supplementary file 1 — (PDF 94 kb) [file 125_2016_3872_MOESM1_ESM.pdf]

Figure S1. Methylation at cg23161492 versus mRNA expression of *ANPEP*.

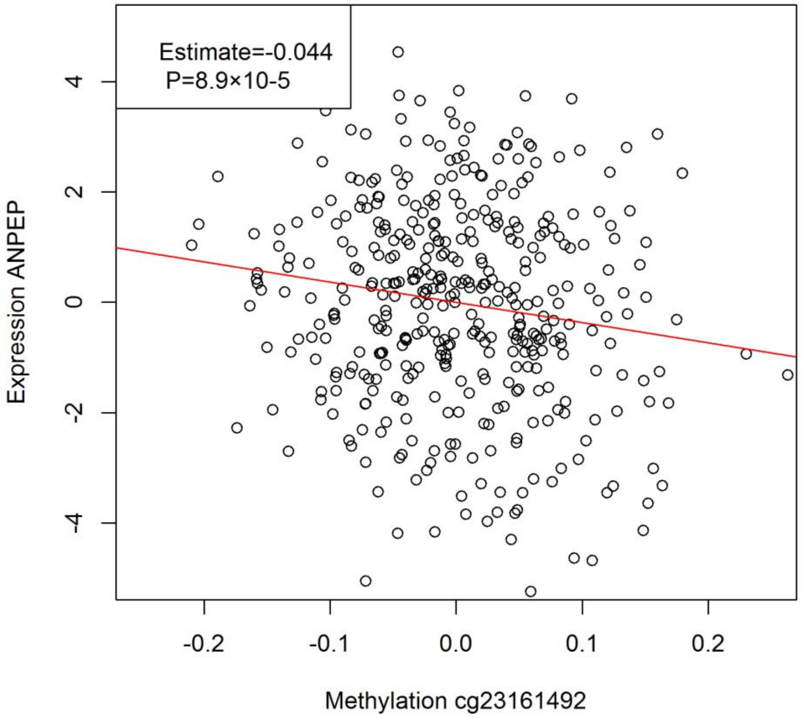

Gene expression is residual expression after adjustment for age, sex, batch effects, measured blood cell counts, fasting state and RNA quality score. DNA methylation is residual methylation after adjustment for age, sex, measured white blood cell counts and batch effects. The estimate is the change in the residuals of gene expression per residual increase of DNA.
