# Supplementary material for: Tobacco smoking is associated with DNA methylation of diabetes susceptibility genes
Source: Diabetologia. 2016 Jan 29;59:998–1006. doi: 10.1007/s00125-016-3872-0 (PMC4826423; doi:10.1007/s00125-016-3872-0)
Supplement: Supplementary file 2 — (PDF 21 kb) [file 125_2016_3872_MOESM2_ESM.pdf]

Table S1. Clinical characteristics of the replication population

		Smoking category			
	**Total**	**Current**	**Former**	**Never**	***P*[a]**
N	674	68	368	238	
Age, years	67.4 (6.0)	66.3 (6.2)	67.7 (5.7)	67.2 (6.2)	0.27
Sex, male (%)	277 (41%)	24 (35%)	176 (48%)	77 (32%)	0.76
Body mass index, kg/m^2	27.5 (4.0)	26.0 (3.5)	28.0 (4.1)	27.1 (3.9)	0.03
Fasting glucose, mmol/l	5.42 (0.57)	5.32 (0.52)	5.49 (0.57)	5.35 (0.56)	0.74
Systolic blood pressure, mmHg	144.7 (22.2)	139.9 (19.4)	145.9 (23.0)	144.3 (21.4)	0.11
Diastolic blood pressure, mmHg	84.5 (11.7)	81.3 (10.0)	85.6 (12.3)	83.9 (11.0)	0.07
Total cholesterol, mmol/l	5.60 (0.99)	5.62 (1.05)	5.59 (1.00)	5.59 (0.97)	0.30
HDL-cholesterol, mmol/l	1.55 (0.44)	1.60 (0.56)	1.52 (0.43)	1.58 (0.42)	0.77
Triglycerides, mmol/l	1.42 (0.79)	1.44 (0.75)	1.50 (0.88)	1.28 (0.60)	0.11
Fasting[b], yes (%)	673 (99.9%)	68 (100%)	367 (99.7%)	238 (100%)	NA

Data are mean (SD) or n (%).
[a]Current versus never smokers.
[b]The subjects who provided blood after an overnight fast.
