# Supplementary material for: Tobacco smoking is associated with DNA methylation of diabetes susceptibility genes
Source: Diabetologia. 2016 Jan 29;59:998–1006. doi: 10.1007/s00125-016-3872-0 (PMC4826423; doi:10.1007/s00125-016-3872-0)
Supplement: Supplementary file 4 — (PDF 7 kb) [file 125_2016_3872_MOESM4_ESM.pdf]

Table S3. Significant associations between tobacco smoking and methylation of diabetes genes, adjusted for potential confounding factors.

CpG site	Beta	Se	*P*	Gene
cg23161492	-0.040	0.006	2.6×10^{-10}	*ANPEP*
cg26963277	-0.025	0.004	2.5×10^{-8}	*KCNQ1*
cg03450842	-0.012	0.003	5.8×10^{-4}	*ZMIZ1*
cg01744331	-0.013	0.003	1.4×10^{-5}	*KCNQ1*
cg16556677	-0.015	0.004	3.8×10^{-5}	*KCNQ1*

Adjusted for age, sex, body mass index, houseman estimated white blood cell proportions, batch effects, systolic blood pressure, total cholesterol, HDL-cholesterol, triglycerides (natural logarithm), alcohol consumption and C-reactive protein (natural logarithm).
