# Supplementary material for: Tobacco smoking is associated with DNA methylation of diabetes susceptibility genes
Source: Diabetologia. 2016 Jan 29;59:998–1006. doi: 10.1007/s00125-016-3872-0 (PMC4826423; doi:10.1007/s00125-016-3872-0)
Supplement: Supplementary file 5 — (PDF 21 kb) [file 125_2016_3872_MOESM5_ESM.pdf]

Table S4. Association between identified CpG sites and expression of nearby genes.

CpG site	Gene	ILMNID	Beta	Se	*P*
cg01744331	*CDKN1C*	ILMN_1718565	4.824	2.044	0.02
cg01744331	*NAP1L4*	ILMN_1804327	0.988	1.096	0.37
cg01744331	*SLC22A18*	ILMN_2382505	-0.005	1.704	1.00
cg16556677	*NAP1L4*	ILMN_1804327	0.775	0.867	0.37
cg16556677	*SLC22A18*	ILMN_2382505	-0.295	1.347	0.83
cg16556677	*CDKN1C*	ILMN_1718565	-0.321	1.623	0.84
cg23161492	*ANPEP*	ILMN_1763837	-4.352	0.973	8.9×10^{-06}
cg23161492	*AP3S2*	ILMN_1731596	0.109	0.35	0.76
cg23161492	*C15ORF38*	ILMN_2189406	-0.111	0.387	0.77
cg26963277	*CDKN1C*	ILMN_1718565	2.998	1.359	0.03
cg26963277	*SLC22A18*	ILMN_2382505	2.007	1.13	0.08
cg26963277	*NAP1L4*	ILMN_1804327	-0.631	0.728	0.39

Residual expression after adjustment for age, sex, batch effects, houseman estimated white blood cell proportions, erythrocytes and platelet cell counts, fasting state and RNA quality score associated with residual methylation after adjustment for age, sex, houseman estimated white blood cell proportions and batch effects.
Estimates are changes in residual expression per percentage residual methylation increase. Bonferroni corrected threshold of significance: $0.05/12=4.2\text{x}10^{-3}$.
