# Supplementary material for: Tobacco smoking is associated with DNA methylation of diabetes susceptibility genes
Source: Diabetologia. 2016 Jan 29;59:998–1006. doi: 10.1007/s00125-016-3872-0 (PMC4826423; doi:10.1007/s00125-016-3872-0)
Supplement: Supplementary file 6 — (PDF 7 kb) [file 125_2016_3872_MOESM6_ESM.pdf]

Table S5. Results for the associations between the replicated CpG sites and fasting serum glucose and insulin levels.

**CpG site**	**Effect**	***P***
fasting glucose		
cg23161492	0.0015	0.58
cg26963277	-0.0012	0.47
cg03450842	-0.0010	0.47
cg01744331	-0.0003	0.79
cg16556677	-0.0006	0.69
fasting insulin		
cg23161492	0.0051	0.10
cg26963277	0.0039	0.04
cg03450842	-0.0002	0.93
cg01744331	0.011	0.45
cg16556677	0.0031	0.09

Effect represents the effect in methylation beta-value per 1-unit increase in fasting glucose (mmol/l) or natural logarithm of insulin (pmol/l). *P* represents the unadjusted p-value.
