# Supplementary material for: Tobacco smoking is associated with DNA methylation of diabetes susceptibility genes
Source: Diabetologia. 2016 Jan 29;59:998–1006. doi: 10.1007/s00125-016-3872-0 (PMC4826423; doi:10.1007/s00125-016-3872-0)
Supplement: Supplementary file 7 — (PDF 20 kb) [file 125_2016_3872_MOESM7_ESM.pdf]

Table S6. Replicated CpG sites, met-QTLs and association with type 2 diabetes.

CpG site	SNP	Distance[a]	Effect Allele	met-QTL beta	met-QTL *P*	T2D OR	T2D *P*
**cg23161492**	rs11073891	6,792	C	0.023	1.5×10^{-14}	1.00	0.88
**cg26963277**	rs231356	17,065	T	-0.010	8.9×10^{-6}	1.06	1.3×10^{-5}
**cg01744331**	rs231356	17,015	T	-0.011	2.5×10^{-6}	1.06	1.3×10^{-5}
**cg16556677**	rs2283194	265	G	-0.011	3.2×10^{-5}	1.00	0.95

[a]Distance between CpG sites and met-QTL SNP.
